# Weight status change from birth to childhood and the odds of high blood pressure among Chinese children

**DOI:** 10.3389/fpubh.2023.1135994

**Published:** 2023-04-06

**Authors:** Cheng Li, Ziqi Liu, Min Zhao, Cheng Zhang, Pascal Bovet, Bo Xi

**Affiliations:** ^1^Department of Epidemiology, School of Public Health, Qilu Hospital, Cheeloo College of Medicine, Shandong University, Jinan, China; ^2^Department of Nutrition and Food Hygiene, School of Public Health, Cheeloo College of Medicine, Shandong University, Jinan, China; ^3^Key Laboratory of Cardiovascular Remodeling and Function Research, Chinese Ministry of Education, Chinese National Health Commission and Chinese Academy of Medical Sciences, The State and Shandong Province Joint Key Laboratory of Translational Cardiovascular Medicine, Department of Cardiology, Qilu Hospital, Cheeloo College of Medicine, Shandong University, Jinan, China; ^4^Center for Primary Care and Public Health, University of Lausanne, Lausanne, Switzerland

**Keywords:** birth weight, childhood weight, weight change, children, high blood pressure

## Abstract

**Background:**

It is well documented that birth weight and childhood weight are associated with the blood pressure (BP) levels in childhood. However, the impact of weight status change from birth to childhood on BP among children is less well described. We aimed to assess the association between changes in weight status from birth to childhood and high BP in childhood.

**Methods and results:**

Data were obtained from a cross-sectional survey conducted in Jinan, China, and a total of 5,546 children aged 6–17 years were included in this study. Based on the birth weight status [high weight (> 4,000 g) vs. normal weight (2,500–4,000 g)] and childhood weight status during the survey period [high weight (overweight and obesity) vs. normal weight], children were assigned into four groups: persistently normal weight (normal birth weight and normal childhood weight), resolved high weight (high birth weight but normal childhood weight), incident high weight (normal birth weight but high childhood weight), and persistently high weight (high birth weight and high childhood weight). After adjustment for sex and age, BP in childhood was more responsive to current body mass index (BMI) than birth weight. After adjustment for the potential covariates, compared with children who had persistently normal weight from birth to childhood, those with incident high weight (odds ratio [*OR*] = 3.88, 95% confidence interval [*CI*] = 3.29–4.57) and persistently high weight (*OR* = 3.52, 95% *CI* = 2.71–4.57) were associated with the increased odds of childhood high BP. However, children who had resolved high weight did not have significantly increased odds of high BP in childhood (*OR* = 0.86, 95% *CI* = 0.59–1.25).

**Conclusion:**

The association of BP with recent BMI was stronger than with birth weight. Children who had incident or persistently high weight from birth to childhood had increased odds of high BP in childhood, whereas the odds was not significantly increased among those with high birth weight but changed to normal weight in childhood. Our findings highlight the importance of maintaining an appropriate weight in the early lifetime for the prevention of high BP and other related diseases, especially for those with high birth weight.

## Introduction

Overweight/obesity has become a serious public issue in the 21st century. The number of overweight/obesity in the world has been on an accelerated increase in the past decades (1). According to data from China Health and Nutrition Study, the relative increase rates of overweight and obesity for children aged 6–17 years from 1991 to 2015 were 358.7 and 621.4%, respectively (2), and the prevalence rate in some areas of China has been comparable to that in developed countries (3). With the overweight/obesity epidemic, the burden of high blood pressure (BP) is also getting heavier, and it is estimated that nearly 1/3 of adults and 1/5 of children and adolescents in China are suffering from high BP (4, 5). Studies showed that hypertensive individuals had begun to develop target organ damages in childhood, such as left ventricular hypertrophy (LVH) and elevated carotid intima-media thickness (cIMT) (6), which were risk factors for cardiovascular diseases (CVD) in adulthood (7, 8). In addition, previous studies suggested the roots of adulthood hypertension can extend back into childhood (9, 10). Thus, the childhood is a critical period to prevent the development and progression of high BP and other related diseases.

Considerable studies have proved that high birth weight or childhood overweight/obesity is associated with childhood high BP (11–13), to our knowledge, however, no studies have examined the relationship between change in weight status from birth to childhood and high BP among children. In addition, although several epidemiological studies have shown that children with overweight who recovered to normal weight had no increased risk of hypertension (14, 15), few studies explored whether reversal from high birth weight to normal childhood weight could reduce the risk of hypertension in childhood. In this study, we aimed to examine the association between weight status change from birth to childhood and high BP in a school-based study of Chinese children.

## Methods

### Study participants

The data were from a cross-sectional survey that carried out from September 2013 to November 2014 in Jinan City, Shandong Province, China, as a part of the “12th Five-Year Plan” National Science and Technology Support Program called “Early Warning, Diagnosis and Treatment of Cardiovascular Diseases in Children.” All participants were selected from four public schools in this city using the convenient cluster sampling method (including two elementary schools, one junior high school, and one senior high school). A total of 7,846 children were initially included in this study, and 2,086 children were excluded because of missing values on BP, weight status or other relevant covariates. Then 5,546 participants were included in this study after further excluding those with low birth weight (<2,500 g, *n* = 108) and childhood underweight (based on the underweight reference for Chinese school-aged children, *n* = 106) (16). Comparison of the demographic characteristics between the included participants and those with data on low birth weight or low childhood weight is summarized in Supplementary Table S1. There was no significant difference between two groups, except for sleep duration. This study was approved by the Ethics Committee of the Capital Institute of Pediatrics in Beijing, China, and we obtained informed consent from all participants and their parents/guardians.

### Demographic information and physical examination

Demographic and lifestyle information such as age, sex, birth weight, bedtime, wake-up time, diet habits (intake of high-sugar foods, fruits, and vegetables) and parental hypertension status were obtained from the self-reported structured questionnaires. Sleep duration was calculated using the following formula: 24 – bedtime + wake-up time. Physical examination was performed by trained staff according to standardized scheme. Children wearing light clothing took off their shoes, and we took the average of two consecutive measurements of weight and height for this analysis, accurate to 0.1 kg and 0.1 cm, respectively. Body mass index (BMI) was calculated through the following formula: BMI (kg/m^2^) = weight (kg)/(height)^2^ (m^2^). After a 10-min rest, the sitting BP was measured using an electronic sphygmomanometer [OMRON HEM-7012, which has been validated in one previous publication (17)] for three consecutive measurements in a quiet environment with suitable light and temperature, with an interval of at least 1 min between each measurement. The fifth Korotkoff phase was recorded as diastolic blood pressure (DBP) and the first as systolic blood pressure (SBP). The BP value measured twice in a row should not exceed 5 mmHg, otherwise the measurement needs to be repeated. And the average of the last two readings was used for data analysis. To make the weight at birth and childhood comparable, we calculated the standardized Z-scores of SBP and DBP for specific age and sex, and categorized Z-scores of SBP or DBP into four groups according to quartiles.

### Definitions of variables of interest

Children with birth weight > 4,000 g was defined as high weight at birth, and those between 2,500 and 4,000 g were defined as normal weight at birth (18). Childhood weight status [high weight (overweight and obesity) vs. normal weight] was defined according to the BMI percentile reference for Chinese children (19). Based on their weight status over the two periods, children were divided into four subgroups: persistently normal weight (normal birth weight and normal childhood weight), resolved high weight (high birth weight but normal childhood weight), incident high weight (normal birth weight but high childhood weight), and persistently high weight (high birth weight and high childhood weight). High BP in childhood was defined as sex-, age-, and height-specific BP ≥ 95th percentile values for Chinese children (20). Sufficient intake of fruits/vegetables was defined as consuming ≥5 servings of fruits/vegetables per day (21) and frequent intake of high-sugar foods was self-defined as consuming high-sugar foods once a day or more because of no relevant reference for support.

### Statistical analysis

The basic characteristics of participants across the weight status change groups were presented as means ± *SD* for continuous variables and numbers (proportions) for categorical variables, and were compared using variance analysis and Chi-square test, respectively. Chi-square test was also used to compare the proportion of weight status across different BP Z-scores groups, and the prevalence of high BP across different weight status change groups. After adjusting for sex and age in childhood, multivariable linear regression was used to estimate the standardized *β* and its 95% confidence interval (*CI*) between BP and birth weight or BMI in childhood. Covariate analysis was performed to compare the BP-Z score across different weight status change groups, and multivariable logistic regression was performed to examine the association between weight status change and childhood high BP. Model 1 was adjusted for sex and age, and model 2 was additionally adjusted for sleep duration, intake of high-sugar foods, and intake of fruits/vegetables. Model 3 was then additionally adjusted for parental hypertension status. Furthermore, we conducted a subgroup analysis by sex and age. All these analyses were performed with SAS version 9.4 and a two-sided *p* value less than 0.05 was considered as statistically significant.

## Results

### Basic characteristic of the participants

A total of 5,546 children (boys: 50.2%) were recruited in this study. Among these children, 53.2 and 7.0% had persistently normal weight and persistently high weight, respectively; 32.7% changed from normal birth weight to high weight in childhood (incident high weight) and 7.1% changed from high birth weight to normal weight in childhood (resolved high weight). There were significant differences in age, sex, birth weight, and the proportion of paternal hypertension across the four weight change groups (Table 1).

**Table 1:** Basic characteristic of participants across the weight status change groups.

	Persistently normal weight (*n* = 2,953)	Resolved high weight (*n* = 396)	Incident high weight (*n* = 1,812)	Persistently high weight (*n* = 385)	Total (*n* = 5,546)	*p*
Sex [*n* (%)]						<0.001
Boys	1,285 (43.5)	232 (58.6)^*^	997 (55.0)^*^	271 (70.4)^*^	2,785 (50.2)	
Girls	1,668 (56.5)	164 (41.4)^*^	815 (45.0)^*^	114 (29.6)^*^	2,761 (49.8)	
Age group [*n* (%)]						
6–9 years	950 (32.2)	110 (27.8)	586 (32.3)	115 (29.9)	950 (32.2)	0.035
10–13 years	1,140 (38.6)	149 (37.6)	744 (41.1)	163 (42.3)	1,140 (38.6)	
14–17 years	863 (29.2)	137 (34.6)	482 (26.6)	107 (27.8)	863 (29.2)	
Birth weight (g)	3,297 ± 333	4,258 ± 327^*^	3,329 ± 327^*^	4,215 ± 277^*^	3,440 ± 459	<0.001
Sleep duration (h/d)	8.5 ± 1.1	8.5 ± 1.2	8.5 ± 1.1	8.6 ± 1.1	8.5 ± 1.1	0.399
Intake of high-sugar foods [*n* (%)]						0.608
Frequent	657 (22.2)	87 (22.0)	381 (21.0)	69 (17.9)	1,194 (21.5)	
Infrequent	2,296 (77.8)	309 (78.0)	1,431 (79.0)	316 (82.1)	4,352 (78.5)	
Intake of fruits/vegetables [*n* (%)]						0.727
Insufficient	203 (6.9)	32 (7.8)	146 (7.9)	27 (6.8)	403 (7.3)	
Sufficient	2,750 (93.1)	396 (92.2)	1,783 (92.1)	383 (93.2)	5,143 (92.7)	
Paternal hypertension [*n* (%)]						0.016
Yes	252 (8.5)	34 (8.6)	204 (11.3)^*^	40 (10.4)	530 (9.6)	
No	2,701 (91.5)	362 (91.4)	1,608 (88.7)^*^	345 (89.6)	5,016 (90.4)	
Maternal hypertension [*n* (%)]						0.236
Yes	81 (2.7)	12 (3.0)	69 (3.8)^*^	13 (3.4)	175 (3.2)	
No	2,872 (97.3)	384 (97.0)	1,743 (96.2)^*^	372 (96.6)	5,371 (96.8)	

Data are presented as mean ± SD for continuous variables and *n* (%) for categorical variables.

* There was a statistical significance between persistently normal weight group and other specific group (*p* < 0.05).

### Association of birth weight and BMI in childhood with BP in childhood

As shown in Table 2, children with different weight status tended to have different BP levels, and those with normal birth weight or normal childhood weight were less likely to have higher BP levels. After adjustment for sex and age in childhood, standardized linear regression showed that the association of SBP Z-score with BMI in childhood (*β*: 0.48, 95% *CI*: 0.46–0.50) was stronger than that with birth weight (*β*: 0.04, 95% *CI*: 0.01–0.06). And BMI in childhood was positively associated with DBP Z-score (*β*: 0.32, 95% *CI*: 0.30–0.35), whereas there was no significant association between birth weight and DBP Z-score (*β*: 0.02, 95% *CI*: −0.002-0.05).

**Table 2:** Association of birth weight and childhood BMI with childhood BP.

	Age- and sex-adjusted BP Z-score in childhood, proportion (95% *CI*)					Standardized β^*^
	Quartile 1	Quartile 2	Quartile 3	Quartile 4	*p* values	
SBP Z-score						
Birth body weight					0.003	0.04 (0.01, 0.06)
Normal (2,500–4,000 g)	88.17 (86.47, 89.87)	86.84 (85.06, 88.62)	83.53 (81.57, 85.48)	85.15 (83.28, 87.02)		
High (≥4,000 g)	11.83 (10.13, 13.53)	13.16 (11.38, 14.94)	16.47 (14.52, 18.43)	14.85 (12.98, 16.72)		
BMI					<0.001	0.48 (0.46, 0.50)
Normal	83.98 (82.05, 85.91)	68.47 (66.02, 70.92)	56.62 (54.01, 59.22)	32.52 (30.05, 34.98)		
Overweight or obesity	16.02 (14.09, 17.95)	31.53 (29.08, 33.98)	43.38 (40.78, 45.99)	67.48 (65.02, 69.95)		
DBP Z-score						
Birth body weight					0.016	0.02 (−0.002,0.05)
Normal (2,500–4,000 g)	88.31 (86.62, 90.00)	84.74 (82.85, 86.64)	84.54 (82.64, 86.44)	86.08 (84.25, 87.90)		
High (≥4,000 g)	11.69 (10.00, 13.38)	15.26 (13.36, 17.15)	15.46 (13.56, 17.36)	13.93 (12.10, 15.75)		
BMI					<0.001	0.32 (0.30, 0.35)
Normal	75.76 (73.50, 78.01)	65.65 (63.15, 68.16)	56.87 (54.26, 59.47)	43.29 (40.68, 45.90)		
Overweight or obesity	24.24 (21.99, 26.50)	34.35 (31.84, 36.85)	43.13 (40.53, 45.74)	56.71 (54.10, 59.32)		

* Standardized β: Standardized linear regression coefficients of birth weight or BMI with BP with adjustment for age and sex.

### Comparison of BP levels and prevalence of hypertension among weight status change groups

**Figure 1:**
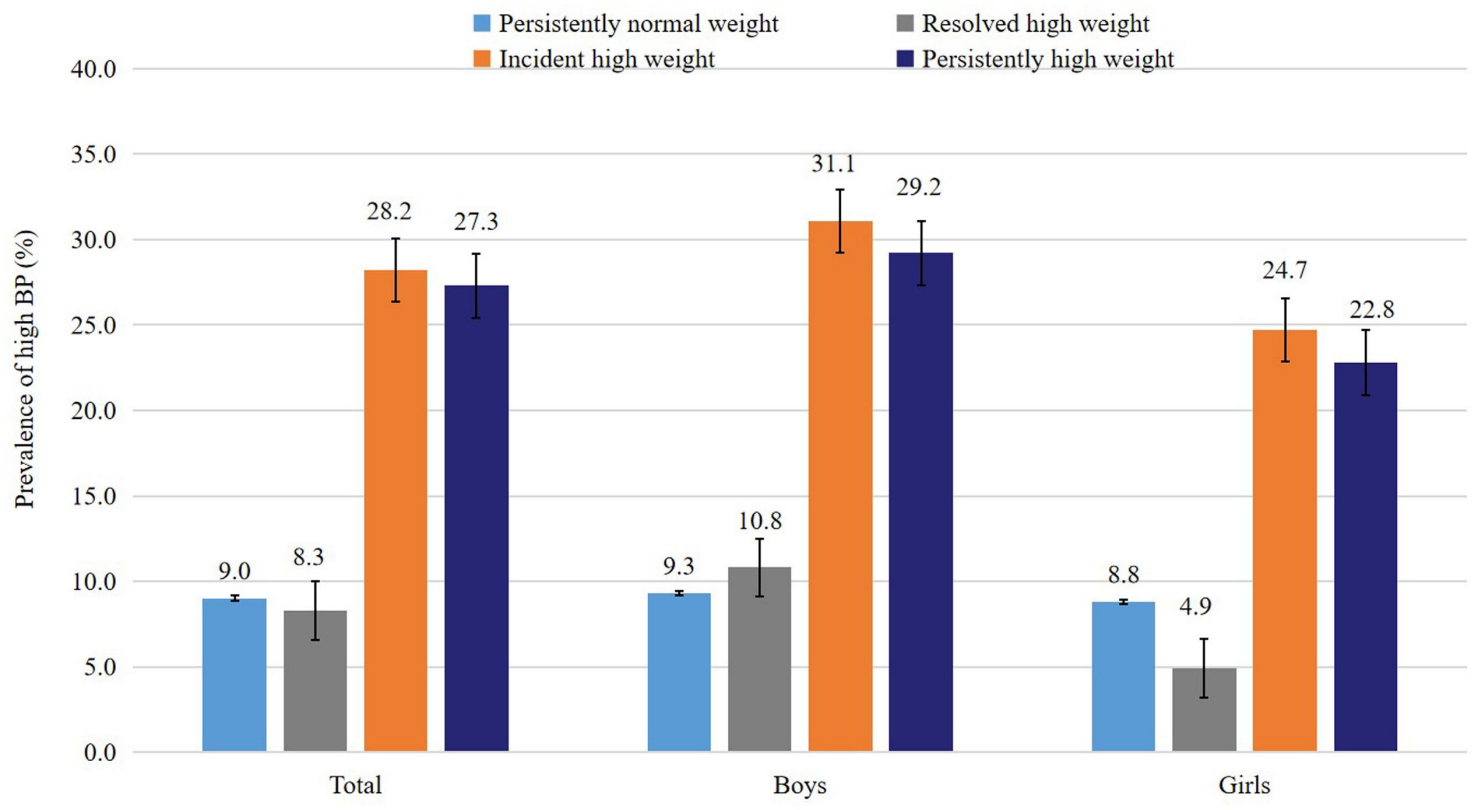
Prevalence of high BP across different weight status change groups.

After adjustment for the covariates, the standardized Z-scores of SBP and DBP of children in incident high weight group (0.48 and 0.32, respectively) and persistently high weight group (0.49 and 0.29, respectively) were significantly higher than those in persistently normal weight group (−0.32 and − 0.21, respectively), whereas the SBP and DBP levels in the resolved high weight group (−0.32 and − 0.17, respectively) were not significantly different from the persistently normal weight group (Table 3). Children who had persistently high weight (27.3%) and incident high weight (28.2%) had a higher prevalence of childhood high BP than those with persistently normal-weight (9.0%) and those with resolved high weight (8.3%; Figure 1). The subgroup analysis by sex and age showed consistent results with the above findings (Table 3).

**Table 3:** Comparison of blood pressure levels and prevalence of high BP among different weight status change groups.

	Persistently normal weight	Resolved high weight	Incident high weight	Persistently high weight	*F*/*χ*^2^	*p*
Total						
SBP *Z*-score	−0.32 (−0.35, −0.28)	−0.32 (−0.41, −0.23)	0.48 (0.44, 0.53) ^*^	0.49 (0.40, 0.58) ^*^	339.4	<0.001
DBP *Z*-score	−0.21 (−0.25, −0.18)	−0.17 (−0.27, −0.08)	0.32 (0.27, 0.36) ^*^	0.29 (0.20, 0.39) ^*^	128.1	<0.001
High BP [*n* (%)]	266 (9.0)	33 (8.3)	511 (28.2) ^*^	105 (27.3) ^*^	352.0	<0.001
Boys						
SBP *Z*-score	−0.41 (−0.46, −0.36)	−0.34 (−0.45, −0.22)	0.48 (0.42, 0.53) ^*^	0.48 (0.37, 0.58) ^*^	219.0	<0.001
DBP *Z*-score	−0.28 (−0.33, −0.22)	−0.17 (−0.30, −0.05)	0.32 (0.26, 0.38) ^*^	0.29 (0.18, 0.41) ^*^	83.4	<0.001
High BP [*n* (%)]	120 (9.3)	25 (10.8)	310 (31.1) ^*^	79 (29.2) ^*^	199.6	<0.001
Girls						
SBP *Z*-score	−0.25 (−0.29, −0.20)	−0.29 (−0.44, −0.15)	0.50 (0.43,0.56) ^*^	0.51 (0.34,0.69) ^*^	132.3	<0.001
DBP *Z*-score	−0.16 (−0.21, −0.11)	−0.17 (−0.32, −0.02)	0.32 (0.25,0.39) ^*^	0.30 (0.12,0.48) ^*^	49.5	<0.001
High BP [*n* (%)]	1,468 (8.8)	8 (4.9)	201 (24.7) ^*^	26 (22.8) ^*^	135.3	<0.001
6–9 years						
SBP *Z*-score	−0.32 (−0.38, −0.26)	−0.28 (−0.46, −0.11)	0.48 (0.40, 0.55) ^*^	0.45 (0.28, 0.62) ^*^	102.4	<0.001
DBP *Z*-score	−0.26 (−0.32, −0.20)	−0.13 (−0.31, 0.04)	0.38 (0.30, 0.45) ^*^	0.33 (0.15, 0.50) ^*^	57.5	<0.001
High BP [*n* (%)]	79 (8.3)	11 (10.0)	166 (28.3)^*^	28 (24.4)^*^	116.1	<0.001
10–13 years						
SBP *Z*-score	−0.34 (−0.40, −0.29)	−0.33 (−0.48, −0.18)	0.50 (0.43, 0.56)^*^	0.44 (0.30, 0.58) ^*^	147.5	<0.001
DBP *Z*-score	−0.23 (−0.29, −0.18)	−0.15 (−0.30, 0.01)	0.32 (0.25, 0.39)^*^	0.30 (0.15, 0.45) ^*^	55.3	<0.001
High BP [*n* (%)]	68 (6.0)	4 (2.7)	169 (22.7)^*^	27 (16.6)^*^	113.6	<0.001
14–17 years						
SBP *Z*-score	−0.28 (−0.34, −0.22)	−0.34 (−0.49, −0.18)	0.47 (0.38, 0.55)^*^	0.59 (0.41, 0.76)^*^	88.1	<0.001
DBP *Z*-score	−0.13 (−0.20, −0.07)	−0.23 (−0.39, −0.07)	0.24 (0.16, 0.33)^*^	0.25 (0.06, 0.43)^*^	19.7	<0.001
High BP [*n* (%)]	119 (13.8)	18 (13.1)	176 (36.5)^*^	50 (46.7)^*^	113.2	<0.001

Adjusted for sex, age, sleep duration, intake of high-sugar foods, intake of fruits/vegetables, and parental hypertension status. BP, blood pressure. Age- and sex-adjusted BP *Z*-scores were presented as mean (95% CI), and prevalence of high BP were presented as *n*(%).

* There was a statistical significance between persistently normal weight group and other specific group (*p* < 0.05).

### Weight status change and childhood hypertension

After adjusting for sex, age, sleep duration, intake of high-sugar foods, intake of fruits/vegetables, and parental hypertension status, compared with children who had persistently normal weight from birth to childhood, those who had persistently high weight [odds ratio (*OR*) = 3.52, 95% *CI* = 2.71–4.57] and incident high weight (*OR* = 3.88, 95% *CI* = 3.29–4.57) had increased odds of high BP in childhood. In contrast, those who had resolved high weight did not have significantly increased odds of high BP (*OR* = 0.86, 95% *CI* = 0.59–1.25). In the subgroup analysis, we observed similar features stratified by age and gender (Table 4).

**Table 4:** Association between weight status change from birth to childhood and high blood pressure among children.

	Model 1		Model 2		Model 3	
	*OR* (95% *CI*)	*P*	*OR* (95% *CI*)	*P*	*OR* (95% *CI*)	*p*
Total						
Persistently normal weight	1.00		1.00		1.00	
Resolved high weight	0.85 (0.58, 1.25)	0.414	0.86 (0.56, 1.25)	0.418	0.86 (0.59, 1.25)	0.420
Incident high weight	3.93 (3.34, 4.63)	<0.001	3.93 (3.34, 4.63)	<0.001	3.88 (3.29, 4.57)	<0.001
Persistently high weight	3.56 (2.74, 4.62)	<0.001	3.55 (2.73, 4.61)	<0.001	3.52 (2.71, 4.57)	<0.001
Boys						
Persistently normal weight	1.00		1.00		1.00	
Resolved high weight	1.08 (0.69, 1.71)	0.732	1.09 (0.69, 1.72)	0.721	1.09 (0.69, 1.72)	0.724
Incident high weight	4.41 (3.50, 5.57)	<0.001	4.42 (3.50, 5.58)	<0.001	4.33 (3.43, 5.47)	<0.001
Persistently high weight	3.94 (2.85, 5.45)	<0.001	3.95 (2.86, 5.47)	<0.001	3.91 (2.82, 5.41)	<0.001
Girls						
Persistently normal weight	1.00		1.00		1.00	
Resolved high weight	0.53 (0.26, 1.11)	0.091	0.54 (0.26, 1.12)	0.097	0.54 (0.26, 1.12)	0.100
Incident high weight	3.46 (2.74, 4.36)	<0.001	3.50 (2.77, 4.43)	<0.001	3.47 (2.75, 4.39)	<0.001
Persistently high weight	3.11 (1.95, 4.98)	<0.001	3.01 (1.93, 4.95)	<0.001	3.09 (1.92, 4.95)	<0.001
6–9 years						
Persistently normal weight	1.00		1.00		1.00	
Resolved high weight	1.20 (0.62,2.34)	0.585	1.20 (0.62,2.33)	0.597	1.21 (0.62,2.36)	0.577
Incident high weight	4.32 (3.23,5.79)	<0.001	4.44 (3.31,5.96)	<0.001	4.46 (3.32,5.98)	<0.001
Persistently high weight	3.46 (2.13,5.63)	<0.001	3.46 (2.13,5.64)	<0.001	3.50 (2.15,5.70)	<0.001
10–13 years						
Persistently normal weight	1.00		1.00		1.00	
Resolved high weight	0.44 (0.16,1.21)	0.111	0.43 (0.16,1.21)	0.111	0.44 (0.16,1.21)	0.112
Incident high weight	4.64 (3.44,6.27)	<0.001	4.63 (3.42,6.25)	<0.001	4.58 (3.39,6.20)	<0.001
Persistently high weight	3.14 (1.93,5.11)	<0.001	3.09 (1.90,5.02)	<0.001	3.08 (1.89,5.01)	<0.001
14–17 years						
Persistently normal weight	1.00		1.00		1.00	
Resolved high weight	0.82 (0.48,1.40)	0.466	0.82 (0.48,1.40)	0.465	0.81 (0.47,1.38)	0.434
Incident high weight	3.34 (2.55,4.38)	<0.001	3.32 (2.53,4.36)	<0.001	3.23 (2.45,4.25)	<0.001
Persistently high weight	4.57 (2.96,7.05)	<0.001	4.56 (2.95,7.06)	<0.001	4.41 (2.84,6.84)	<0.001

Model 1: Adjusted for sex and age. Model 2: Model 1 + additionally adjusted for sleep duration, intake of high-sugar foods, and intake of fruits/vegetables.

Model 3: Model 2 + additionally adjusted for parental hypertension status.

OR: odds ratio; CI: confidence interval.

## Discussion

Our study suggested that although birth weight was positively associated with BP in childhood, its association with BP in childhood was weaker than BMI in childhood. And children who had incident high weight or persistently high weight from birth to childhood had higher odds of childhood high BP than those who had persistently normal weight. However, those with high birth weight but transition to normal weight in childhood did not have higher odds of high BP in childhood.

It is well-documented that the early lifetime is a sensitive period for the promotion of cardiovascular health in children (22–24). Previous studies had shown that weight change in early life was associated with the risks of CVD events, one of which was high BP (25, 26). For instance, a birth cohort study including 1,119 adolescents (mean age: 10.2 years) showed that SBP and DBP in adolescence increased by 1.17 mmHg (95% *CI* = 0.69–1.66) and 1.03 mmHg (95% *CI* = 0.59–1.47) with one standard deviation increase of weight-for-age-Z-score from birth to 5 years, respectively (27). The data from Boston-area pre-birth cohort showed that each additional increment of BMI-Z-score during the first 6 postnatal months and in the preschool years was associated with 0.81 (95% *CI* = 0.15–1.46) and 1.61 (95% *CI* = 0.33–2.89) mmHg higher SBP of children aged 6–10 years, respectively (28). Consistent with those findings above, our results showed that children who had weight gain or remained high weight from birth to childhood had higher BP levels and odds of high BP in childhood.

Several previous studies showed that reversing high weight status during childhood can decrease the risk of childhood hypertension (14, 15). For instance, a prospective study including 6,512 adolescents aged 12–14 years in China showed that compared with those who had normal weight at both baseline and follow-up, those who had incident overweight (*OR* = 3.73, 95% *CI* = 2.49–5.59) or persistently overweight (*OR* = 4.83, 95% *CI* = 3.66–6.37) had a higher risk of childhood high BP, whereas those who were overweight at baseline but normal weight at follow-up did not have such risk (*OR* = 1.36, 95% *CI* = 0.71–2.60) (14). In addition, reversing the weight status from childhood to adulthood can also decrease the risk of hypertension in adulthood. One prospective study with a median follow-up of 11 years showed that compared with participants who were normal weight in both childhood and adulthood, those who were overweight in adulthood irrespective of their weight status in childhood had an increased risk of adult hypertension (29). Inversely, participants with overweight in childhood but changed to normal weight in early adulthood did not have the increased risk of adult hypertension (29). One meta-analysis also showed that those who reversed overweight status in childhood to normal weight status in adulthood had no significant difference in the risk of hypertension in adulthood compared with those who had persistently normal weight (*OR* = 1.25, 95% *CI* = 0.73–2.13) (30). But all those studies above ignored the association of birth weight with childhood BP, while our research filled in this gap. Our results showed that reversing high weight status from birth to childhood (high birth weight to normal childhood weight) was able to decrease the odds of high BP in childhood. In addition, another important finding of our study is that we demonstrated that the BP was more responsive to recent weight than birthweight in early life. One school-based cohort study in the Seychelles also showed that weight change during any period since birth was associated with BP in childhood, and the association of BP with recent weight was stronger than that with early weight in life, which was consistent with ours (25). Those findings combined with ours highlighted the importance of maintaining a normal weight during childhood, especially for children who had high birth weight in early life. And children who had normal birth weight should also be noted that if being overweight/obese in childhood, the risk of hypertension will also increase.

High BP was a major risk factor for CVD events and previous studies evaluating the relationship between weight status change and CVD events showed similar features with our findings. For example, one meta-analysis showed that compared with participants who had persistently normal weight from childhood to adulthood, the increased odds of CVD in adulthood was found in those with incident overweight (*OR* = 2.76, 95% *CI* = 1.79–4.27) and persistently overweight (*OR* = 3.04, 95% *CI* = 1.69–5.46), but the odds was not significantly increased among those with reversed overweight (*OR* = 1.22, 95% *CI* = 0.92–1.62) (30). As for risk factors for CVD, children who were resolved high weight during childhood had no significant difference in the risk of childhood high cIMT with those who had persistently normal weight during childhood (31). In addition, individuals whose weight status changed from overweight in childhood to normal weight in adulthood did not have the increased odds of high cIMT (32), LVH (33), dyslipidemia (30), and type 2 diabetes (34) in adulthood. Therefore, these findings further suggest that early intervention for overweight/obese individuals may have long-term beneficial association with cardiovascular health in children.

Although we are the first to assess the association between changes in weight status from birth to childhood and high BP among children, there are also several limitations that needed to be mentioned. First, children’s birth weight is primarily derived from parental recollections, so recall bias may exist. But previous research has shown that parents’ memories of birth weight are reliable (35). Second, the study subjects are only from Jinan urban areas, so the generalizability of the results may be limited. Third, data on parental hypertension status, and children’s sleep duration and diet habits were obtained from a self-reported questionnaire, thus there might be recall and information biases. Fourth, we did not collect data on puberty status and gestation age, which may influence the results. Fifth, this study is based on a cross-sectional design, so future longitudinal study is needed to further validate our findings. The weight status during childhood shows a dynamic process, thus examining the effect of BMI trajectory patterns on childhood BP in a longitudinal study is of great significance, especially for children with overweight/obesity.

## Conclusion

Our results indicated that the BP during childhood was more responsive to recent weight than birthweight, and the adverse association of BP with high birth weight can be attenuated or even limited by reversing to a normal weight later in life. These findings highlight the importance of maintaining an appropriate weight in the lifetime to prevent high BP and other related diseases, especially for children with high birth weight.

## Data availability statement

The raw data supporting the conclusions of this article will be made available by the authors, without undue reservation.

## Ethics statement

This program was approved by the Ethics Committee of the Capital Institute of Pediatrics in Beijing, China. Written informed consent to participate in this study was provided by the participants’ legal guardian/next of kin.

## Author contributions

BX designed the study and was the principal investigator. ZL performed the data analysis. CL drafted the first version of the manuscript. BX, MZ, CZ, and PB critically revised the manuscript. All authors contributed to the article and approved the submitted version.

## Funding

This study was funded by the National Natural Science Foundation of China (81673195).

## Conflict of interest

The authors declare that the research was conducted in the absence of any commercial or financial relationships that could be construed as a potential conflict of interest.

## Publisher’s note

All claims expressed in this article are solely those of the authors and do not necessarily represent those of their affiliated organizations, or those of the publisher, the editors and the reviewers. Any product that may be evaluated in this article, or claim that may be made by its manufacturer, is not guaranteed or endorsed by the publisher.
