# Supplementary material for: Weight status change from birth to childhood and the odds of high blood pressure among Chinese children
Source: Front Public Health. 2023 Apr 6;11:1135994. doi: 10.3389/fpubh.2023.1135994 (PMC10116612; doi:10.3389/fpubh.2023.1135994)
Supplement: Supplementary file 1 [file Table_1.DOC]

**Table S1 Characteristics of the included participants and those with data on low birth weight or low childhood weight**

|  | Participants with low birth weight or low childhood weight (n=214) | Included participants in this study (n=5546) | *t*/χ2 | *P* |
| --- | --- | --- | --- | --- |
| Sex [n (%)] |  |  | 0.37 | 0.543 |
| Boys | 112 (52.3) | 2785 (50.2) |  |  |
| Girls | 102 (47.7) | 2761 (49.8) |  |  |
| Age group [n (%)] |  |  | 3.56 | 0.169 |
| 6-9 years | 81 (37.9) | 1761 (31.8) |  |  |
| 10-13 years | 76 (35.5) | 2196 (39.6) |  |  |
| 14-17 years | 57 (26.6) | 1589 (28.7) |  |  |
| Sleep duration (h/d) | 8.7 ± 1.1 | 8.5 ± 1.1 | 2.46 | 0.014 |
| Intake of high-sugar foods [n (%)] |  |  | 0.02 | 0.88 |
| Frequent | 47 (22.0) | 1194 (21.5) |  |  |
| Infrequent | 167 (78.0) | 4352 (78.5) |  |  |
| Intake of fruits/vegetables [n (%)] |  |  | 0.44 | 0.509 |
| Insufficient | 13 (6.1) | 403 (7.3) |  |  |
| Sufficient | 201 (93.9) | 5143 (92.7) |  |  |
| Paternal hypertension [n (%)] |  |  | 0.01 | 0.918 |
| Yes | 20 (9.4) | 530 (9.6) |  |  |
| No | 194 (90.7) | 5016 (90.4) |  |  |
| Maternal hypertension [n (%)] |  |  | 0.01 | 0.924 |
| Yes | 7 (3.3) | 175 (3.2) |  |  |
| No | 207 (96.7) | 5371 (96.8) |  |  |

Data are presented as mean ± *SD* for continuous variables and n (%) for categorical variables.
